# Practice of Non-Institutional Delivery and Its Associated Factors Among Women Who Gave Birth in Southern Ethiopia, 2022

**DOI:** 10.1089/whr.2023.0005

**Published:** 2023-07-12

**Authors:** Temesgen Geta Hardido, Fekire Sugebo Woshimato, Fekadu Anjulo Nasero

**Affiliations:** ^1^ School of Nursing, College of Medicine and Health Sciences, Wolaita Sodo University, Wolaita, Ethiopia.; ^2^ School of Nursing, College of Medicine and Health Sciences, Wachemo University, Hossana, Ethiopia.

**Keywords:** Boloso Bombe, Woreda, Southern Ethiopia, non-institutional delivery, delivered women

## Abstract

**Background:**

Since 2000, the Ethiopian ministries of health and other stakeholders have taken some measures to enhance institutional delivery. However, the Ethiopian Demographic Health Survey 2019 report indicated that more than 50% of Ethiopian reproductive-age women gave birth outside health facilities. Therefore, the purpose of this study was to assess the practice of noninstitutional delivery among women who gave birth at Boloso Bombe Woreda (district) in southern Ethiopia.

**Methods:**

A community-based cross-sectional study was carried out on 252 study participants from June to July 2022 in Boloso Bombe Woreda. Data collection was done using a structured questionnaire and systematic sampling techniques were used to select the study subjects. Data were entered into the EPI data, version 3.1, and analyzed using SPSS, version 25. Adjusted odds ratios (AORs), along with 95% confidence intervals (CIs), were used and the level of statistical significance was declared at a *p*-value of 0.05.

**Results:**

In this study, 252 participants completed the survey, with a 97% response rate. The prevalence of noninstitutional delivery among study participants was 68.7% (95% CI: 63.1–72.9). In this study, mother's occupation, such as working as a daily laborer (AOR = 3.6; 95% CI [1.2–11.2]); absence of antenatal care history (AOR = 3.3; 95% CI [1.3–8.6]); poor knowledge of labor complications (AOR = 3.5; 95% CI [2.2–6.1]); and place of first delivery (AOR = 8.7; 95% CI [3.2–23]) were factors that were positively and significantly associated with the practice of noninstitutional delivery. However, last pregnancy planned was negatively associated with the practice of noninstitutional delivery (AOR = 0.4; 95% CI [0.2–0.8]).

**Conclusions:**

This study indicated that the majority of study participants practiced noninstitutional delivery in this study area. Therefore, we strongly recommend that all responsible bodies should take immediate action to reduce the practice of noninstitutional delivery and improve those identified factors.

## Introduction

In Africa, only about half of the 123 million women who give birth each year receive antenatal care (ANC), neonatal care, and delivery care. To promote maternal, newborn, and child health, women must have access to basic health care facilities during childbirth.^1^ The World Health Organization (WHO) reports that in high-income countries, more than 90% of women give birth at a health center, while in low-income countries, most women give birth outside a health center with the assistance of an untrained person.^2^ Noninstitutional delivery means just giving birth outside a health institution and in the home with the assistance of an unskilled person.^3^

Many women, no matter where they give birth, have similar problems after they give birth. These problems include dizziness, tender abdomen, blood loss, fetal death, and uterine rupture. However, these problems are often considered critical if a woman delivers the baby outside a health facility.^4^ Most maternal mortality (MM) occurs during the delivery and in the immediate postpartum period.

All women should have access to basic maternity care during their pregnancy, such as clean and safe delivery. They should also have access to emergency obstetric care if necessary.^5^ In 2017, due to noninstitutional deliveries, health facilities, and health care provider-related factors, 462 and 11 children per 100,000 live births died in developed and developing countries, respectively.^6^

In addition, women giving birth outside a health institution are at a threefold higher risk of developing complications. In addition, women giving birth outside a health institution are at higher risk of death.^7^ The rate of neurological dysfunction and seizure for noninstitutional delivery is three times higher compared with institutional delivery.^8^ Increasing delivery rates in the health institution is a crucial approach to prevent the death of a mother and her baby.

However, maintaining the practice of institutional delivery has not consistently changed into reduced maternal death in low- and middle-income countries.^9^ Although there are some improvements in reduction of maternal and infant mortality across the world, sub-Saharan African countries and southern Asia accounted for the majority of (86%) estimated world MM in 2017; of this, 2/3 of MM belonged to the sub-Saharan African countries only.^4,6^

Ethiopia is 1 of the 15 countries considered to be “very high alert” or “high alert,” being a fragile state. Practicing institutional delivery is the key approach that prevents 13%–33% and 20%–30% of maternal and neonatal mortality, respectively.^10^ Although the Ethiopian government and nongovernmental organizations have tried to stop women from giving birth outside health facilities, 50% of Ethiopian women have given birth outside these facilities.^11^

To reduce noninstitutional delivery utilization, factors that limit access to institutional delivery must be identified and addressed at health system and societal levels.^12^ The noninstitutional delivery rate varies from region to region. In addition, no previous study was done in this study area. Therefore, this study was aimed at assessing the practice of noninstitutional delivery among women who gave birth in the study area.

## Methodology

### Study area, period, and design

A cross-sectional study was carried out from June to July 2022 in the Boloso Bombe Woreda. The district is located about 57 km from the town of Wolaita zone, southern Ethiopia, and 435 km from Addis Ababa, the capital city of Ethiopia. The geographical location of Boloso Bombe is 70 1′ 32″-70 11′ 30″ N latitude and 370 26′ 18″-370 39′ 38″ E longitude.

According to the Boloso Bombe Woreda base plan report of 2013, the Woreda has a total population of 114,342. Of these, 57,400 were females and 56,942 were males and there were 26,642 reproductive-age women. The district has 21 small administrative units (Kebeles) and one primary hospital, 4 health centers, and 8 health posts.

### Population and eligibility criteria

Only women who had given birth in the past 6 months and lived in the selected area for at least 1 year were included in the study population. Mothers who did not give full information on the required variables, were unable to hear and speak, and had any mental problems were not included in this study.

### Sample size determination and its procedures

A sample size of 252 participants was determined using a single population proportion. Five percent margin of error, 95% confidence interval (CI), 19% noninstitutional delivery from the previous study, and 10% nonresponse were considered to calculate the final sample size.^13^

According to the WHO recommendation, 30% (7) Kebeles were selected from the total Kebeles by using lottery methods. The total number of mothers who delivered in the selected Kebeles from February 2021 to February 2022 was determined using immunization registration books and health posts in family folders. The sample size in each selected Kebele was proportionally allocated to the total number of deliveries in each Kebele. Then, respondents were recruited using a systematic sampling method. Every other respondent in each Kebele was interviewed.

### Operational definitions

#### Nonutilization of institutional delivery

Women who delivered their last baby outside a health facility with the assistance of nonskilled or traditional attendants were classified as those who did not utilize institutional delivery services.^14^

### Knowledge of labor complications

Those who mentioned greater than or equal to three labor complications were classified as having good knowledge,^15^ and those who mentioned less than three complications were assumed to have poor knowledge.^15^

### Data collection and analysis

A structured questionnaire, including four parts, was used to collect data on sociodemographic information, obstetric-related factors for women, health care provider-related factors, and knowledge of labor complications. The last component comprised one question with a list of top six labor complications adapted from a previous study.^15^ Women who mention at least three knowledge questions are considered to have good knowledge. Women who mention less than three knowledge questions are considered to have poor knowledge. These four components and their items were independent variables, and the place of last delivery was the dependent variable.

Home-to-home visits and face-to-face interviews were used to collect data. A structured questionnaire was adapted from the previous study.^15^ Data were collected by seven BSc nurses and supervised by three health officers. One-day training was given to the data collectors and supervisors on the procedures of data collection. The questionnaire was prepared in English, then translated into the local language by experts, and again translated into English to enhance the consistency. During data collection, data collectors and supervisors were closely supervised by the principal investigator to maintain data consistency and completeness.

The completeness and consistency of data were checked, coded, and entered into EPI Data, version 3.1. For analysis, the data were exported to SPSS, version 25. To present descriptive statistics, frequencies and percentages, means and standard deviations, tables, and pie charts were used. The crude odds ratio with its 95% interval was calculated using a binary logistic regression test to test for associations between dependent and independent variables. Then, variables with *p* < 0.25 in the bivariate analysis were taken as candidates for the multivariate analysis.

Finally, the multivariate analysis with adjusted odds ratio (AOR) was used to control possible confounders and to determine predictors of prevalence of noninstitutional delivery. A *p*-value of <0.05 was considered statistically significant.

## Results

### Sociodemographic features

A total of 252 respondents completed the interview with a response rate of 97%. The mean age of the study participants was 28.07 years with a standard deviation of 5.3 years. The age range of study participants was between 15 and 29 years. One hundred twenty-two (48.4%), 205 (81.3%), 217 (86.3%), and 176 (69.8) participants in the study were housewives, were married, had finished high school, and belonged to the Wolaita ethnic group, respectively (Table 1).

**Table 1.** Sociodemographic Characteristics of the Respondents at Boloso Bombe Woreda in Southern Ethiopia, 2022 (*n* = 252)

Sociodemographic characteristics	Frequency	%
Respondents' age, years		
15–29	140	55.6
30–40	83	32.9
41–49	29	11.5
Respondents' educational level		
Cannot read and write	42	16.7
Can read and write	59	23.4
Primary school	39	15.9
Secondary school and above	112	44.4
Respondents' occupation		
Housewife	122	48.4
Merchant	73	29
Civil servant	37	14.7
Daily laborer	20	7.9
Partners' education		
Cannot read and write	17	6.7
Can read and write	76	30.2
Primary school	64	25.4
Secondary school and above	95	37.7
Partners' occupation		
Farmer	114	45.2
Merchant	82	32.5
Civil servant	45	17.9
Others^a^	11	4.4
Respondents' ethnicity		
Wolaita	176	69.8
Gamo-Gofa	22	8.2
Kambata	41	16.3
Amhara	11	4.3
Others^b^	6	2.4
Respondents' religion		
Orthodox	69	27.4
Muslim	56	22.2
Protestant	78	31
Others^c^	49	19.4
Respondents' marital status		
Married	205	81.3
Single	16	6.3
Others^d^	31	12.3
Decision maker		
Woman	145	57.5
Husband	107	42.5
Household head		
Woman	53	21
Husband	199	79

^a^ Private employee, unemployed, student, or daily laborer.

^b^ Oromo, Hadiya, or Gurage.

^c^ Apostolic, Catholic, or Joba.

^d^ Widowed or divorced.

### Obstetric- and provider-related factors affecting respondents

As for obstetric variables, most of the participants (93.7%) reported having had between 0 and 3 abortions. A total of 196 study participants (77.8%) participated in ANC follow-up during their last pregnancy and 198 (78.6%) planned the pregnancy before the birth of their last child. In addition, 127 (50.4%) participants had a history of 1–3 pregnancies and 138 (55.2%) made a joint decision. In addition, 42.9% of them had 1–3 children. One hundred ninety (75.4%) participants had no abortion history and only 16 of them had a history of more than or equal to 4 abortions. The remaining 46 participants had a history of 1–3 abortions.

Regarding provider-related variables, this finding revealed that 73 (21%) respondents did not receive respectful care during the previous delivery and 85 (34.5%) respondents reported that their privacy was not maintained during a past delivery. On the other hand, poor belief in health facilities (36.4%), sudden onset of labor (16.8%), and no respect from health care providers (23.2%) were the three major reasons that contributed to noninstitutional delivery.

### Respondents' knowledge of obstetric complications

Among the 252 participants, around 60% of women had poor knowledge and 40% had good knowledge of obstetric complications (Table 2).

**Table 2.** Obstetric- and Health Care Provider-Related Factors Affecting the Respondents at Boloso Bombe Woreda in Southern Ethiopia, 2022 (*n* = 252)

Gravidity		
1–3	127	50.4
4–6	94	37.3
>6	31	12.3
Parity		
1–3	108	42.9
4–6	97	38.2
>6	47	18.7
Abortion		
0	190	75.4
1–3	46	18.3
≥4	16	6.3
ANC follow-up		
Yes	196	77.8
No	56	22.2
Last pregnancy planned		
Yes	198	78.6
No	54	21.4
Place of previous (past) delivery		
Noninstitutional	107	42.6
Institutional	141	57.4
Place of current delivery		
Noninstitutional	173	68.7
Institutional	79	31.3
Knowledge of labor and delivery complications		
Good	150	59.5
Poor	102	40.5
Wanted the current delivery place		
Yes	173	68.7
No	79	31.3
Obstetric complications of last pregnancy		
Yes	140	55.6
No	112	44.4
Decision maker on last delivery		
Woman and her husband	138	55.2
Husband	107	41.7
Woman	6	2.4
Health professional	2	0.8
Friendly behavior by the provider		
Yes	190	63.5
No	62	36.5
Respectful care		
Yes	179	79
No	73	21
Privacy maintained		
Yes	165	65.5
No	85	34.5
Reasons for noninstitutional delivery		
I have not seen any advantage of HI delivery	20	11.5
The health professional did not intentionally attend to the delivery	12	6.9
Sudden onset of labor	29	16.8
No respect from the service provider	40	23.2
Poor belief on the institution	63	36.4
Long distance	9	5.2

ANC, antenatal care; HI, health institution.

### Prevalence of noninstitutional delivery

Among the total study participants, 68.7% practiced noninstitutional delivery and 31.3% practiced institutional delivery (Table 2).

### Factors affecting the practice of noninstitutional delivery

Findings from this study indicated that mother's occupation, such as working as a daily laborer (AOR = 3.6; 95% CI [1.2–11.2]); last pregnancy planned (AOR = 0.4; 95% CI [0.2–0.8]); no history of ANC follow-up (AOR = 3.3; 95% CI [1.3–8.6]); poor knowledge of labor complications (AOR = 3.5; 95% CI [2.2–6.1]); and place of past delivery (AOR = 8.7; 95% CI [3.2–23]) were statistically associated with the outcome variable (Table 3).

**Table 3.** Factors Associated with Noninstitutional Delivery Among Respondents at Boloso Bombe Woreda in Southern Ethiopia, 2022 (*n* = 252)

Factors	COR	***p***	AOR (CI)	***p***
Respondents' occupation				
Daily laborer	0.46 (0.13–1.23)	0.27	3.6 (1.2–11.2)	0.024
Merchant	0.3 (0.1–0.7)	0.11	4.3 (1.4–13.1)	0.010
Housewife	0.2 (0.1–0.69)	0.07	2.0 (0.6–6.8)	0.26
Civil servant	1	1	1	1
Last pregnancy planned				
No	1.9 (1.5–5.7)	0.08	0.4 (0.2–0.8)	0.002
Yes	1	1	1	1
Last pregnancy ANC follow-up				
No	0.24 (0.13–0.44)	0.00	3.3 (1.3–8.6)	0.015
Yes	1	1	1	1
Complications occurred at the last delivery				
Yes	4 (2.4–7.4)	0.00	2.5 (1.2–5.2)	0.010
No	1	1	1	1
Place of past delivery				
Home	0.2 (0.1–0.9)	0.21	8.7 (3.2–23)	0.000
Health post	2 (1.2–6.2)	0.14	2.5 (1.0–6.0)	0.000
HC and hospital	1	1	1	1
Knowledge of labor and delivery complications				
Poor knowledge	0.4 (0.2–0.72)	0.06	3.5 (2.2–6.1)	0.011
Good knowledge	1	1	1	1

AOR, adjusted odds ratio; CI, confidence interval; COR, crude odds ratio; HC, health center.

## Discussion

The rate of noninstitutional delivery in the study was 68.7%. This finding is higher than in studies done in the South Wollo Zone, Delanta District (35.2%)^16^; Nepal (41.9%)^17^; and Brazil (11.7%).^18^ Findings from the study are in line with the study done in Afar (71%).^19^ However, the variation in both cases may be due to differences in sociodemographic status, sample size and study period, geographic location, and methodological variation. A study done in eastern Africa using Demographic Helath Survey indicated that noninstitutional delivery was highest among Ethiopian women (72.5%), which is in line with this study.

This consistency might be due to similarity in sociodemographic characteristics and geographic location. However, it indicated a lower value in the other East African countries, such as Tanzania (34.7%), Kenya (37%), Mozambique (2.8%), Rwanda (6.8%), and Malawi (7.1%).^20^ Possible reasons for the discrepancies could be differences in study participant characteristics, quality of delivery services, policies and strategies, research areas, sampling methods, and participant sample size.

In this study, respondents' occupational status was significantly associated with noninstitutional delivery; mothers who were daily laborers were 3.6 times more likely to give birth in noninstitutions than mothers who were civil servants. This study is supported by studies done in Gambela^14^ and Benishangul Gumuz.^21^ This may be due to the following: being a daily laborer can inhibit easy access to health-related information, such as advantages of institutional delivery and/or disadvantages of noninstitutional delivery; they may easily be exposed to economic problems that might inhibit access to a health facility; they may be more exposed to family pressure and cultural influences; and they may not have enough time to reach a health facility.

Place of previous delivery was significantly associated with noninstitutional delivery. Women who gave their last birth outside a health center were 8.7 times more likely to give birth outside a health center than their counterparts. This study was in line with studies done in Ethiopia.^22,23^ The reason can be due to mothers lacking adequate information on the advantages of institutional deliveries and considering them as less risk for complications if they gave birth outside a health institution.

The study found that women with no ANC follow-up were 3.3 times more likely to give birth outside a health center than those with ANC follow-up, which is consistent with studies done in Delanta District,^16^ Zala Woreda,^24^ and Nigeria.^19^ This may be because mothers who attend ANC visits have a chance to find out the importance of institutional delivery.

On the other hand, study participants who had no previous knowledge of labor and delivery complications were 3.5 times more likely to give birth outside a health center than their control group. The reason for this could be that these women did not know the outcomes of a difficult labor and were not looking for health care from the hospital. Complications that occurred at the previous delivery were identified as explanatory variables significantly associated with noninstitutional delivery.

Respondents who had complications at the last delivery were 2.5 times more likely to give birth outside health facilities compared with their counterparts. The possible explanation might be occurrence of complications that can contribute to stress, dissatisfaction, and limit the utilization of institutional delivery.

Finally, a planned pregnancy was negatively and significantly associated with nonutilization of institutional delivery; mothers who planned their last pregnancy were 60% less likely to practice noninstitutional delivery compared with their counterpart group. This may be because mothers who planned their last pregnancy may have an interest in seeking health care, following health care recommendations, and cooperating with their partners.

## Conclusions

In general, 68.7% of mothers have given birth outside a health care facility. The main reasons for this are poor belief in the health care provider and the sudden onset of labor, as well as a lack of respect from health care providers. Respecting mothers and providing health education are two of the most strongly recommended actions that health providers must take. In addition, the district health office and zonal health department should facilitate ambulance services and a maternal waiting area to prevent the sudden onset of labor.

In addition, respondents' occupation, last planned pregnancy, place of previous birth, ANC follow-up of last pregnancy, and knowledge of delivery and birth complications were relevant factors. Health care extension workers, health care providers, and other stakeholders should raise women's awareness of the benefits of ANC follow-up and pregnancy planning and complications associated with home birth.

Finally, zonal health departments and top health institution managers should monitor, evaluate, and supervise the utilization of delivery services provided at each facility.

## Data Availability

The data collected and/or analyzed in the current study are not available to the public before publication to prevent any misuse by the public, but are available upon reasonable request from the corresponding author.

## Declarations

The authors declare that this article is their original work and has never been presented in any University and they understand that plagiarism will not be tolerated and all directly quoted material has been appropriately referenced.

## Abbreviations Used

ANC: antenatal care
AOR: adjusted odds ratio
CI: confidence interval
COR: crude odds ratio
HC: health center
MM: maternal mortality
WHO: World Health Organization

## References

[1] Mustafa S. UNFPA, Maternal Health in Africa: Fact Sheet. 2013. Available from: https://www.unfpa.org/sites/default/files/resource-pdf/EN_Maternal%20Health%20in%20Africa_factsheet_web.pdf [Last accessed: June 2, 2022].

[2] World Health Organization. Maternal Mortality, Key Fact Sheet. 2017. Available from: https://www.paho.org/en/topics/maternal-health [Last accessed: June 2, 2022].

[3] James KM. Motherhood: Home Birth Vs. Hospital Birth. 2016. Available from: https://www.mothermag.com/home-birth-vs-hospital-birth/ [Last accessed: June 3, 2022].

[4] Ryoko S, Yoshito T. Advantages and disadvantages of institutional delivery and home delivery: A qualitative study in Northern Nigeria. Eur J Prev Med 2021;9(1):19–24; doi: 10.11648/j.ejpm.20210901.14

[5] Anna J. Why are there low institutional delivery rates in the gambia? women's opinion. University of Oslo. 2007. Available from: https://core.ac.uk/download/pdf/30858827.pdf [Last accessed: June 5, 2022].

[6] World Health Organization. Maternal Mortality; Key Fact Sheets. 2019. Available from: https://communitymedicine4all.com/2019/09/20/who-updates-fact-sheet-on-maternal-mortality-19-september-2019 [Last accessed: June 2, 2022].

[7] American Associates. Ben-Gurion University of the Negev. (2019, March 4). Home births are three times more dangerous than hospital deliveries. ScienceDaily. Available from: www.sciencedaily.com/releases/2019/03/190304095841.htm [Last accessed: April 10, 2023].

[8] Melinda R. WebMD, What to know about home delivery. 2021. Available from: https://www.webmd.com/baby/what-to-know-about-home-birth [Last accessed: June 2, 2022].

[9] Anna D. In low- and middle-income countries, is delivery in high quality obstetric facilities geographically feasible? Health Affairs 2019;38(9):1576–1584. 31479351 10.1377/hlthaff.2018.05397

[10] Moyer RM. Adanu RM, Cyril E. The relationship between facility-based delivery and maternal and neonatal mortality in Sub-Saharan Africa Chery. Int J Gynaecol Obstet 2013;122(3):263–265; doi: 10.1016/j.ijgo.2013.04.010 23809657

[11] Central Statistical Agency. Ethiopian Demographic health survey. 2016. Addis Ababa. Available from: https://dhsprogram.com/pubs/pdf/FR328/FR328.pdf [Last accessed: June 4, 2022].

[12] USAID. Ethiopia Fact Sheet Maternal and Child Health. 2016. Available from: https://2017-2020.usaid.gov/sites/default/files/documents/Ethiopia-Fact-Sheet_Maternal-Child-Health_Oct-2020.pdf [Last accessed: June 5, 2022].

[13] Gultie T, Wasihun B, Kondale M, et al. Home delivery and associated factors among reproductive age women in Shashemene Town, Ethiopia. J Womens Health Care 2016;5:1; doi: 10.4172/2167-0420.1000300

[14] Gora GA, Umer MF, Ojulu PO, et al. Non-institutional childbirths and the associated socio-demographic factors in Gambella Regional State, Ethiopia. Int J Environ Res Public Health 2021;18(6):2859. 33799658 10.3390/ijerph18062859 PMC8001352

[15] Asmelash AM, Abraham LD. Determinants of home delivery among mothers in Abobo District, Gambella Region, Ethiopia: A case control study. Int J Reprod Med 2020;2020:8856576, 7 pages; doi: 10.1155/2020/8856576 PMC7787860 33490230

[16] Wodaynew T, Fekecha B, Abdisa B. Magnitude of home delivery and associated fac-tors among antenatal care booked mothers in Delanta District, South Wollo Zone, North East Ethiopia: A cross-sectional study. Int J Womens Health Wellness 2018;4:086; doi: 10.23937/2474-1353/1510086

[17] Dhakal P, Shrestha M, Baral D, et al. Factors affecting the place of delivery among mothers residing in Jhorahat VDC, Morang, Nepal. Int J Commun Based Nurs Midwifery 2018;6(1):2. PMC5747568 29344531

[18] Wachs LS, Nunes BP, Soares MU, et al. Prevalence of home care and associated factors in the Brazilian elderly population. Cad Saude Publica 2016;32(3):e00048515. 27027455 10.1590/0102-311X00048515

[19] Abdella M, Abraha A, Gebre A, et al. Magnitude and associated factors for home delivery among women who gave birth in last 12 months in Ayssaita, Afar, Ethiopia-2016. A community based cross sectional study. Glob J Fertil Res 2017;2(1):30–39.

[20] Lemma DR, Assefa T, Biruk ST. Prevalence and associated factors of home delivery in Eastern Africa: Further analysis of data from the recent Demographic and Health Survey data. SAGE Open Med 2022;10:1–10; doi: 10.1177/20503121221088083 PMC8949735 35342629

[21] Berhe R, Nigusie A. Magnitude of home delivery and associated factors among child bearing age mothers in Sherkole District, Benishangul Gumuz regional state-Western-Ethiopia. BMC Public Health 2020;20:796; doi: 10.1186/s12889-020-08919-8 32460736 PMC7251823

[22] Shabeza AI, Tilahun B. Analyzing prevalence of home delivery and associated factors in Anlemo District, Southern Ethiopia. Int Ann Med 2017;1(5); doi:10.24087/IAM.2017.1.6.169

[23] Fira AA, Tariku TB. Delivery site preferences and associated factors among married women of child bearing age in Bench Maji Zone, Ethiopia. Ethiop J Health Sci 2016;26(1):45–54; doi: 10.4314/ejhs.v26i1.9 26949316 PMC4762959

[24] Kucho B, Mekonnen N. Delivery at home and associated factors among women in child bearing age, who gave birth in the preceding two years in Zala Woreda, southern Ethiopia. J Public Health Epidemiol 2017;9(6):177–188.

